# Deciphering the function and evolution of the target of rapamycin signaling pathway in microalgae

**DOI:** 10.1093/jxb/erac264

**Published:** 2022-06-17

**Authors:** Manuel J Mallén-Ponce, María Esther Pérez-Pérez, José L Crespo

**Affiliations:** Instituto de Bioquímica Vegetal y Fotosíntesis, Consejo Superior de Investigaciones Científicas-Universidad de Sevilla, Sevilla, Spain; Instituto de Bioquímica Vegetal y Fotosíntesis, Consejo Superior de Investigaciones Científicas-Universidad de Sevilla, Sevilla, Spain; Instituto de Bioquímica Vegetal y Fotosíntesis, Consejo Superior de Investigaciones Científicas-Universidad de Sevilla, Sevilla, Spain; Max Planck Institute of Molecular Plant Physiology, Germany

**Keywords:** Chlamydomonas, microalgae, nutrient, red algae, TOR kinase

## Abstract

Microalgae constitute a highly diverse group of photosynthetic microorganisms that are widely distributed on Earth. The rich diversity of microalgae arose from endosymbiotic events that took place early in the evolution of eukaryotes and gave rise to multiple lineages including green algae, the ancestors of land plants. In addition to their fundamental role as the primary source of marine and freshwater food chains, microalgae are essential producers of oxygen on the planet and a major biotechnological target for sustainable biofuel production and CO_2_ mitigation. Microalgae integrate light and nutrient signals to regulate cell growth. Recent studies identified the target of rapamycin (TOR) kinase as a central regulator of cell growth and a nutrient sensor in microalgae. TOR promotes protein synthesis and regulates processes that are induced under nutrient stress such as autophagy and the accumulation of triacylglycerol and starch. A detailed analysis of representative genomes from the entire microalgal lineage revealed that the highly conserved central components of the TOR pathway are likely to have been present in the last eukaryotic common ancestor, and the loss of specific TOR signaling elements at an early stage in the evolution of microalgae. Here we examine the evolutionary conservation of TOR signaling components in diverse microalgae and discuss recent progress of this signaling pathway in these organisms.

## Teaser

TOR, the master regulator of cell growth, is conserved in all eukaryotes, including the highly diverse microalgae. We review current knowledge about the conservation and regulation of TOR signaling in microalgae.

## Introduction

Eukaryotic unicellular algae (hereafter microalgae) constitute a highly diverse group of aquatic photosynthetic organisms that are present in almost all ecosystems, ranging from oceans and freshwater to extreme environments such as snow, hot springs, and acidic waters. Together with photosynthetic prokaryotes (cyanobacteria), microalgae are thought to generate about half of the oxygen produced on the planet (Chapman, 2013). In addition to this fundamental biological role, microalgae nowadays represent a valuable natural source of multiple compounds, from biofuels to pharmaceutical products, cosmetics, food and feed, and new materials for high-tech manufacture (Brodie *et al.*, 2017). Indeed, microalgae display unique features for biotechnological and environmental applications as they efficiently harvest sunlight and do not require complex growth conditions. Microalgae can grow in salt- and/or polluted waters, and are used in environmental biotechnology to mitigate CO_2_ emissions and wastewater treatment, among many other applications.

**Fig. 1.**
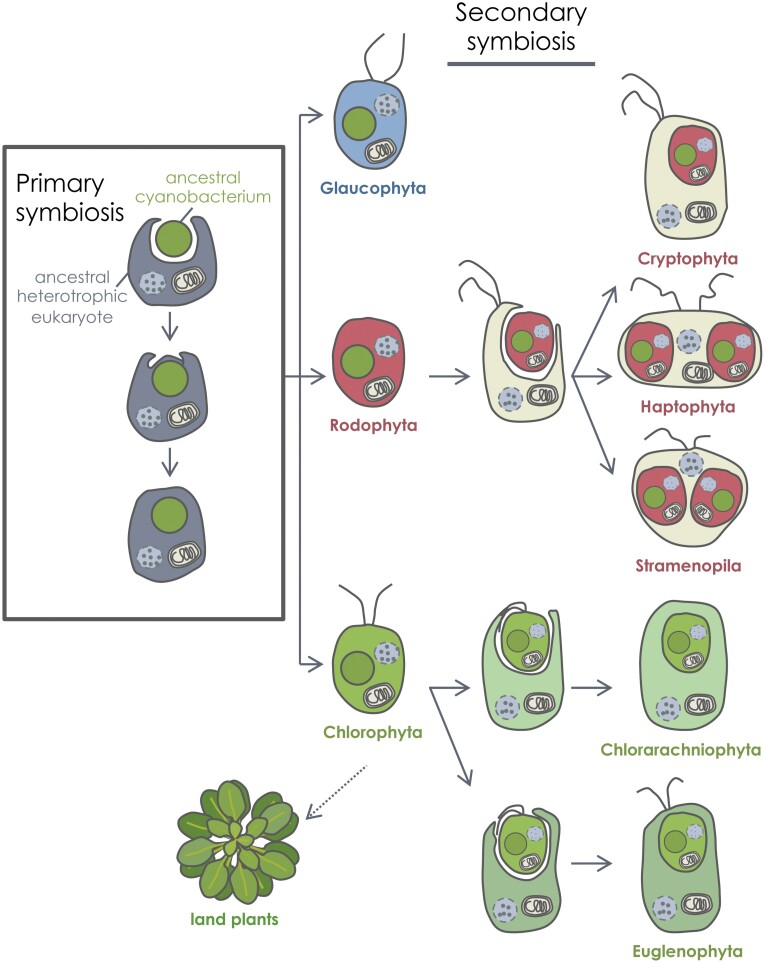
Schematic representation of the origin and evolution of microalgae by primary and secondary endosymbiosis. A single primary endosymbiosis between an ancestral cyanobacterium and an ancestral heterotrophic eukaryote led to the groups of Glaucophyta, Rodophyta, and Chlorophyta. Subsequently, plastids extended to other eukaryotes from chlorophytes and rodophytes by eukaryote to eukaryote endosymbiotic events. On the one hand, a single secondary endosymbiosis between a Rodophyta ancestor and a heterotrophic host led to cryptophytes, haptophytes, and stramenopiles. On the other hand, two different secondary endosymbiosis events from two Chlorophyta ancestors and two unrelated hosts led to the lineages of Chlorarachniophyta and Euglenophyta.

The origin of microalgae traces back to the engulfment of an ancient cyanobacterial lineage by an unknown heterotrophic eukaryotic cell, the so-called ‘primary’ endosymbiosis that led to the first photosynthetic eukaryote ~1.6–2.1 billion years ago (Strassert *et al.*, 2021). Subsequently, a massive gene transfer from the cyanobacterium to the host nucleus took place, generating a primary plastid with a smaller cyanobacterial-derived genome. Primary endosymbiosis gave rise to the three lineages of Archaeplastida: Chlorophyta (green algae), Rhodophyta (red algae), and Glaucophyta (Fig. 1). However, current photosynthetic biodiversity is not due solely to this primary endosymbiosis, and genetic, biochemical, and ultrastructural evidence indicates that many eukaryotic algae acquired plastids through ‘secondary’ endosymbiosis (Archibald and Keeling, 2002; Reyes-Prieto *et al.*, 2007; Tirichine and Bowler, 2011). In such endosymbiotic events, a eukaryote obtained a plastid by engulfing a photosynthetic eukaryote with a primary plastid. In most cases, the primary algal cell involved in secondary endosymbiosis was a red alga, giving rise to a wide variety of photosynthetic eukaryotes including cryptophytes, haptophytes, and stramenopiles (Fig. 1). Major lineages containing red algal-derived plastids include microalgae of global ecological importance in modern oceans such as diatoms (Stramenopila) and *Emiliania huxleyi* (Haptophyta). Green algae have also generated two algal lineages through secondary endosymbiosis with unrelated hosts, the relatively rare marine Chlorarachniophyta, and Euglenida (Fig. 1), which are common microalgae in marine and freshwater environments. Thus, the complex evolution of microalgae has placed these fascinating organisms among the most diverse and primitive eukaryotes (Strassert *et al.*, 2021).

All eukaryotes, including microalgae, have developed sophisticated mechanisms coupling cell growth to changing nutrient conditions. The target of rapamycin (TOR) kinase is a clear example of a highly conserved eukaryotic protein that promotes cell growth when nutrients are available. TOR signaling has been well characterized in yeasts and mammals, but our current knowledge about this pathway in microalgae is still limited (Perez-Perez *et al.*, 2017; Pancha *et al.*, 2020). In this review, we discuss the evolutionary conservation and nutrient regulation of TOR signaling in diverse microalgae.

## Evolutionary conservation of TOR complex components in microalgae

The TOR kinase is an essential regulator of cell growth that integrates nutrient signals with the cell growth machinery. TOR was originally identified in the budding yeast *Saccharomyces cerevisiae*, and subsequent studies in mammals and other organisms indicated that this protein is highly conserved from yeast to humans (for reviews, see Wullschleger *et al.*, 2006; Soulard *et al.*, 2009; Loewith and Hall, 2011; Laplante and Sabatini, 2012). TOR associates with other proteins to assemble two structurally distinct complexes, termed TOR complex 1 (TORC1) and TOR complex 2 (TORC2), which define two independent signaling branches (Loewith *et al.*, 2002). The core components of TORC1 include the TOR kinase, LST8, and Raptor/Kog1, whereas TORC2 contains TOR, LST8, Sin1/Avo1, and Rictor/Avo3. Besides TOR, LST8 is the only protein shared by TORC1 and TORC2. LST8 is essential for TOR activity since it interacts with the kinase domain of TOR, and this binding is needed for full catalytic activity of both TORC1 and TORC2 (Kim *et al.*, 2003; Wullschleger *et al.*, 2005; Aylett *et al.*, 2016). The functional specificity of TORC1 and TORC2 is determined by the presence of Raptor or Rictor in the complex. These two proteins seem to play basic roles in the proper assembly and stabilization of TOR complexes as well as substrate recognition.

**Fig. 2.**
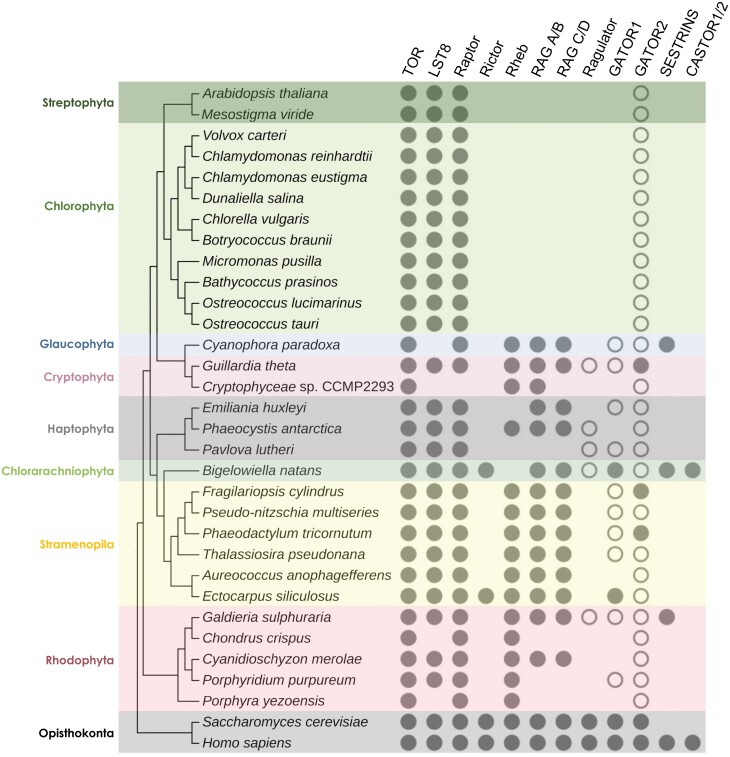
Evolutionary conservation of core components and regulators of the TOR signaling pathway in microalgae. The opisthokonts *Homo sapiens* and *Saccharomyces cerevisiae* and the streptophytes *Arabidopsis thaliana* and *Mesostigma viride* were included as reference. Protein sequences were obtained from the NCBI (ncbi.nlm.nih.gov), JGI (phycocosm.jgi.doe.gov), or directly from the specific genome database using the Orthofinder software. The cladogram was constructed based on 18S rRNA sequences of the indicated species. Sequences were identified using the SILVA rRNA database and aligned using the MUSCLE algorithm in MEGA-X. Filled circles represent conservation while open circles indicate partial conservation of some of the proteins that make up the Ragulator or GATOR1/2 complexes.

The TORC1 core proteins TOR, LST8, and Raptor are found in nearly all eukaryotes, including microalgae and their closest relatives the land plants (Fig. 2). Therefore, it is assumed that TORC1 was found in the last eukaryotic common ancestor (LECA) and has been conserved during the course of eukaryotic evolution (van Dam *et al.*, 2011; Tatebe and Shiozaki, 2017). The presence of TORC1 proteins in ancient microalgae from Rhodophyta and Chlorophyta indicates an early evolutionary origin of this signaling pathway in photosynthetic eukaryotes. Curiously, no evident LST8 proteins are found in some microalgae including the Glaucophyta *Cyanophora paradoxa* and some rhodophytes (Fig. 2). An even more atypical evolution of TORC1 can be found in the Cryptophyta *Cryptophyceae* sp. CCMP2293, which seems to lack both LST8 and Raptor but retains TOR (Fig. 2). Given the essential role of these proteins in TOR function, it is plausible that these microalgae contain functional orthologs of LST8 and Raptor, although this possibility has not been explored yet.

Unlike TORC1, TORC2 does not seem to be conserved in microalgae. It has been proposed that TORC2 emerged in the LECA but was lost at an early stage in the evolution of microalgae and plants (van Dam *et al.*, 2011; Tatebe and Shiozaki, 2017). This hypothesis is supported by the presence of a protein with homology to Rictor in the ancient Chlorarachniophyta *Bigelowiella natans*. The genetic and cellular complexity of *B. natans* is unusual as this microalga retained a relict endosymbiont nucleus, the nucleomorph, which might require intricate coordination of different cellular compartments (Curtis *et al.*, 2012). Rictor is also present in the Stramenopila *Ectocarpus siliculosus*, a brown alga closely related to the red microalga *Aureococcus anophagefferens* (Fig. 2). Thus, it is plausible that some microalgae may retain the capacity to assemble a TORC2-like complex, although it might be challenging to demonstrate this hypothesis.

In mammals and yeasts, the small GTPases Rheb and RAGs regulate TORC1 (Gonzalez and Hall, 2017; Wolfson and Sabatini, 2017). These proteins are not conserved in green microalgae (Chlorophyta) and plants (Streptophyta), which evolved from green microalgae ~700 million years ago (Morris *et al.*, 2018), but are found in many other photosynthetic eukaryotes including glaucophytes, rhodophytes, cryptophytes, haptophytes, chlorarachniophytes, and stramenopiles (Fig. 2). The presence of Rheb and RAGs in such diverse microalgae suggests that these proteins arose with TORC1 in LECA but were lost during evolution in the green algae lineage. Other well-defined upstream regulators of TORC1 in yeasts and mammals such as Ragulator, GATOR1/2, SESTRINS, and CASTOR1/2 (Gonzalez and Hall, 2017; Wolfson and Sabatini, 2017) are overall missing in green algae and plants but present irregularly in some microalgae (Fig. 2). Remarkably, two nucleomorph-containing microalgae, the Chlorarachniophyta *B. natans* and the Cryptophyta *Guillardia theta*, are among the group of microalgae that conserve some of these TORC1 regulators (Fig. 2).

## TORC1 architecture in microalgae

**Fig. 3.**
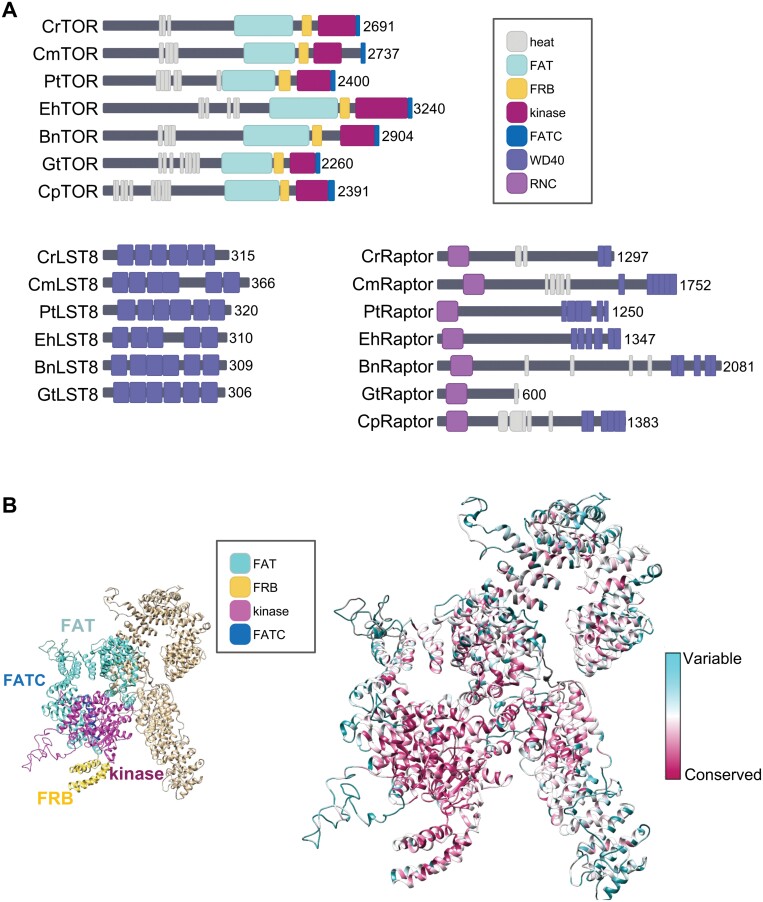
Schematic representation of the domain structure of core TORC1 components from microalgae. (A) TOR, LST8, and Raptor proteins from representative microalgae showing the main conserved domains. The number of amino acids for each protein is indicated on the right. The accession numbers and species abbreviations are as follows: Cr, *Chlamydomonas reinhardtii* (TOR: Cre09.g400553; LST8: Cre17.g713900; Raptor: Cre08.g371957); Cm, *Cyanidioschyzon merolae* (TOR: CMR018CT; LST8: CMH260CT; Raptor: CMH109CT); Pt, *Phaeodactylum tricornutum* (TOR: Phatr21660; LST8: Phatr36142; Raptor: Phatr18549); Eh, *Emiliania huxleyi* (TOR: EOD29434; LST8: EOD08181; Raptor: EOD38139); Bn, *Bigelowiella natans* (TOR: JGI_V11_87739; LST8: JGI_V11_92596; Raptor: JGI_V11_37264); Gt, *Guillardia theta* (TOR: EKX46017; LST8: EKX54172; Raptor: EKX33243);and Cp, *Cyanophora paradoxa* (TOR: g13353.t1; LST8: not identified; Raptor: g20528.t1). (B) Comparative model structure of Chlamydomonas TOR kinase showing the conserved FAT, FRB, kinase, and FATC domains (left panel), and the conserved (pink) and variable (blue) amino acids in microalgal TOR proteins (right panel). Functional protein domains were predicted using the SMART EMBL tool, and amino acid conservation was analyzed using the ConSurf server interface and visualized using Chimera software.

Cryo-electron microscopy studies revealed that the three core proteins of TORC1 in mammals are arranged in a hollow rhomboid dimer where TOR occupies a central position and LST8 and Raptor contribute peripheral parts of the complex (Aylett *et al.*, 2016; Yang *et al.*, 2017). These studies also showed that TORC1 is dimeric and contains two copies of each subunit arranged in a ‘ying–yang’ manner (for a recent review, see Tafur *et al.*, 2021). This particular structure of TORC1 depends on the presence of distinctive domains in the mTOR kinase highly conserved throughout evolution. TOR is a large protein (~280 kDa) that contains at its N-terminus a number of HEAT (Huntington, EF3A, ATM, TOR) repeats followed by FAT (FRAT, ATM, TRRAP), FRB (FKBP12-rapamycin binding), kinase, and FATC (FAT C-terminal) domains (Fig. 3A). All these domains play important roles in the structural conformation and function of TORC1 (Tafur *et al.*, 2021). The HEAT repeats fold into a structure that interacts with the contiguous HEAT domains from the other monomer in the complex, whereas the FRB and FATC domains are part of the catalytic site of the kinase domain. The FAT domain links conformational changes that take place at the N-terminus of the protein to the kinase domain.

The domain architecture of TOR is conserved in all groups of microalgae although the size of the protein can slightly fluctuate (Fig. 3A). A variable number of canonical HEAT repeats are found at the N-terminus of TOR proteins, ranging from three repeats in the model green microalga *Chlamydomonas reinhardtii* (hereafter Chlamydomonas) to 11 detected in the Glaucophyta *Cyanophora paradoxa*. FAT, FRB, kinase, and FATC domains can also be identified in microalgal TOR proteins (Fig. 3A). The conservation of all these domains suggests that TOR might form a dimeric complex in microalgae similar to mammalian TORC1. To date, the only experimental evidence showing the presence of TOR in high molecular mass complexes in photosynthetic eukaryotes comes from biochemical studies performed in Chlamydomonas (Diaz-Troya *et al.*, 2008a). Gel filtration and sucrose density gradient assays indicated that TOR localizes in a 1–2 MDa complex. Moreover, biochemical fractionation and immunofluorescence microscopy studies revealed that TOR and LST8 associate with microsomal membranes that are enriched in the peri-basal body region of Chlamydomonas cells (Diaz-Troya *et al.*, 2008a).

The presence of TOR and LST8 proteins in a rapamycin-sensitive TOR complex similar to TORC1 has been shown in Chlamydomonas (Diaz-Troya *et al.*, 2008a). As reported for yeasts and mammals, the Chlamydomonas LST8 protein interacts with the kinase domain of TOR, suggesting that it might play an essential role in promoting TOR function in microalgae. LST8 is composed entirely of WD40 repeats, which fold into a seven-bladed β-propeller. This domain arrangement is well conserved in LST8 proteins from different microalgae (Fig. 3A). Raptor is the defining component of TORC1 and has different functions including the regulation of TORC1 assembly and the recruitment of kinase substrates in mammals (Hara *et al.*, 2002; Kim *et al.*, 2002). Like TOR, Raptor is a multidomain protein consisting of an RNC (Raptor N-terminal Conserved) domain, several HEAT repeats, and a C-terminal β-propeller. Software prediction indicates that these domains are conserved in evolutionarily distant microalgae, with the exception of the Cryptophyta *Gillardia theta*, which has a significantly smaller Raptor protein (Fig. 3A).

The presence of well-conserved domains in TOR, LST8, and Raptor from microalgae (Fig. 3A) suggests that these proteins may assemble in a complex similar to mammalian TORC1. Indeed, protein modeling and conservation analyses predict that important structural features in TORC1 such as the FRB domain and the LST8-binding region, which regulate the kinase activity of TOR, are evolutionarily conserved in microalgae (Fig. 3B). On the other hand, the absence of Rictor in most microalgae and plants suggests that TORC2 is not structurally conserved in photosynthetic eukaryotes. It is possible, however, that microalgae and plants developed a TOR complex functionally similar to TORC2 but structurally composed of proteins highly different from Rictor. Nevertheless, the precise structure and protein composition of TOR complex(es) in microalgae remain to be determined.

## Dissecting the TORC1 signaling pathway in microalgae

The sensitivity of TORC1 to rapamycin has been crucial to functionally dissect this signaling pathway in yeasts and mammals. The effectiveness of this drug to inhibit cell growth by targeting TORC1 has been tested only in a few microalgae. Rapamycin inhibits, but does not fully arrest, cell growth of Chlamydomonas (Crespo *et al.*, 2005). Lower sensitivity to rapamycin has been reported in the diatom *Phaeodactylum tricornutum* and the photosynthetic protist *Euglena gracilis* (Mukaida *et al.*, 2016; Prioretti *et al.*, 2017). In contrast, *Cyanidioschyzon merolae*, an established model red microalga (Miyagishima and Tanaka, 2021; Pancha *et al.*, 2021), displays complete resistance to this TOR inhibitor (Imamura *et al.*, 2013). The different sensitivity of microalgae to rapamycin most probably relies on the capacity of the FKBP12 protein to bind this drug rather than on structural modifications in TOR. This hypothesis is supported by the findings that expression of yeast FKBP12 in *C. merolae* confers sensitivity to rapamycin (Imamura *et al.*, 2013), and point mutations that increase the affinity of Chlamydomonas FKBP12 for rapamycin provide higher growth susceptibility to this drug (Crespo *et al.*, 2005).

The null to moderate sensitivity of microalgae to rapamycin is in line with the high tolerance of land plants to this drug. The vegetative growth of the model plant *Arabidopsis thaliana* is insensitive to rapamycin (Menand *et al.*, 2002) although at high concentrations the drug can retard root and leaf growth (Menand *et al.*, 2002; Xiong and Sheen, 2012; Xiong *et al.*, 2013). The development of new ATP-competitive TOR-specific inhibitors such as AZD8055 or Torin1 has contributed to dissecting the TOR signaling pathway in plants, although these two inhibitors do not fully mimic the effect of rapamycin (for further information on the different effects of TOR inhibitors, see Montane and Menand, 2019). In this section, we will discuss recent progress in the function and regulation of the TOR signaling pathway in microalgae. The study of the plant TOR pathway is beyond the scope of this review, and the interested reader is referred to recent reviews on this topic (Dobrenel *et al.*, 2016a; Schepetilnikov and Ryabova, 2018; Fu *et al.*, 2020; Ingargiola *et al.*, 2020; Mugume *et al.*, 2020; Burkart and Brandizzi, 2021; Artins and Caldana, 2022; Liu and Xiong, 2022).

Early studies in yeasts demonstrated that TOR inactivation by rapamycin elicits a nutrient starvation response characterized by the accumulation of storage molecules, the activation of autophagy, and the inhibition of protein synthesis, among other metabolic effects (Loewith and Hall, 2011). A similar response has been reported in microalgae upon TOR inhibition, indicating a conserved role for TOR in these organisms. To date, TOR signaling in microalgae has been investigated mainly in Chlamydomonas and *C. merolae*. Mounting evidence indicates that TOR regulates cell growth in microalgae by promoting anabolic processes such as translation and inhibiting catabolic processes such as autophagy. The use of TOR inhibitors has been instrumental in dissecting TOR signaling in microalgae. Treatment of Chlamydomonas cells with rapamycin resulted in the inhibition of protein synthesis (Diaz-Troya *et al.*, 2011), a well-established process downstream of TOR. As previously reported in yeast and mammals (reviewed in De Virgilio and Loewith, 2006; Ma and Blenis, 2009), TOR might promote protein synthesis in Chlamydomonas by activating translation initiation and ribosome biogenesis.

**Fig. 4.**
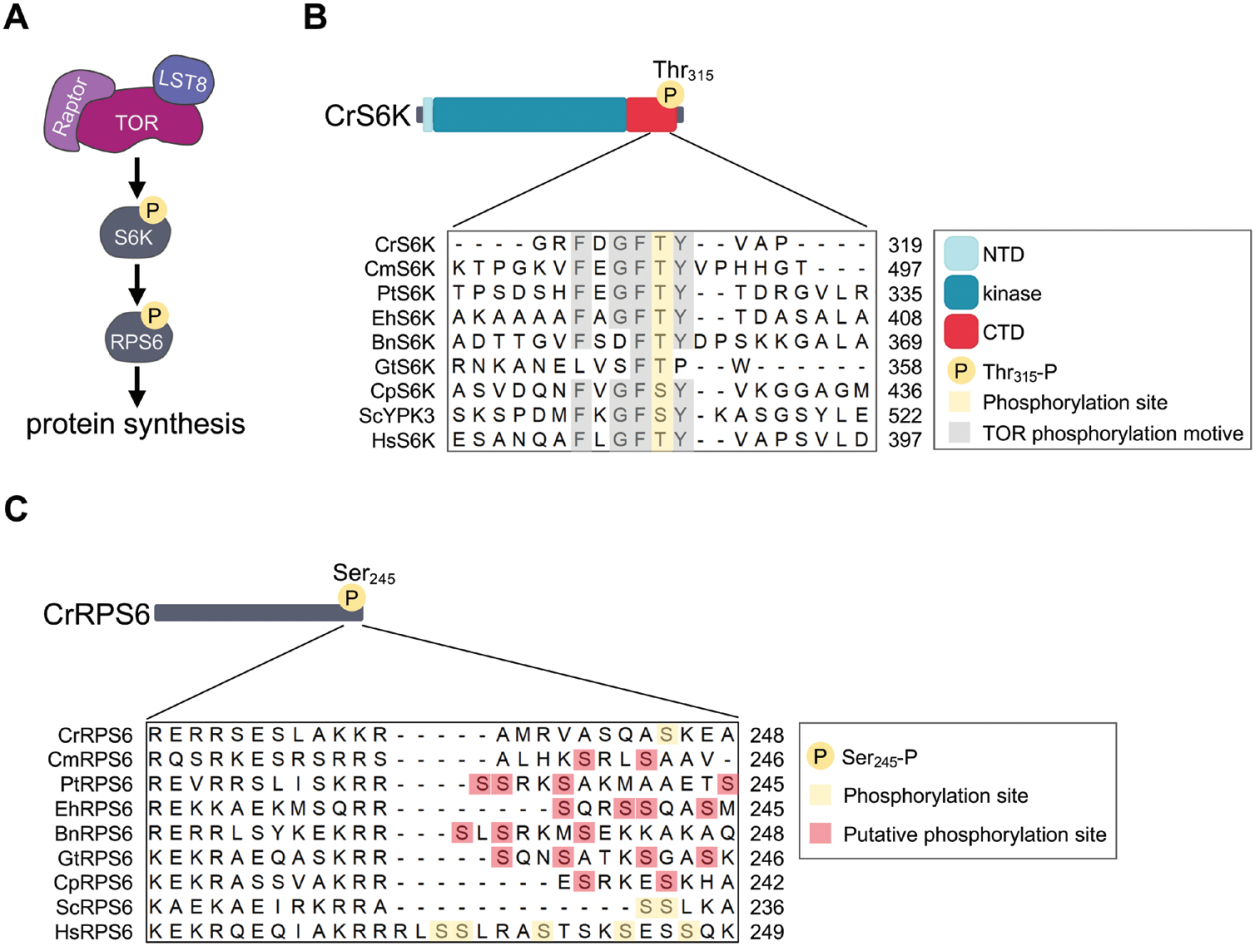
Conservation of TOR-regulated phosphorylation in S6K and RPS6 proteins from microalgae. (A) Schematic representation of the TORC1–S6K–RPS6 signaling branch. In humans, TORC1 phosphorylates S6K at Thr389, which in turn phosphorylates RPS6 to activate protein synthesis. (B) Schematic representation of Chlamydomonas S6K (CrS6K) showing the conserved domains and the phosphorylated Thr315. NTD, N-terminal domain; CTD, C-terminal domain. Sequence alignment of the C-terminal domain of S6K proteins from different microalgae, yeasts, and humans. The conserved phosphorylated Thr is highlighted in yellow, whereas surrounding conserved residues from the TOR phosphorylation motif are labeled in gray. (C) Chlamydomonas RPS6 (CrRPS6) protein showing the phosphorylated Ser245. Sequence alignment of the C-terminus of RPS6 proteins from different microalgae, yeast, and human. TOR-regulated phosphorylation of Ser residues is highlighted in yellow, whereas putative phosphorylatable Ser residues are shown in red. The position of the last amino acid shown in the alignment is indicated for S6K and RPS6 proteins. The accession numbers and species abbreviations are as follows: Cr, *Chlamydomonas reinhardtii* (S6K: Cre13.g579200; RPS6: Cre09.g400650); Cm, *Cyanidioschyzon merolae* (S6K: CMR193CT; RPS6: CMT154CT); Pt, *Phaeodactylum tricornutum* (S6K: Phatr8996; RPS6: Phatr18559); Eh, *Emiliania huxleyi* (S6K: EOD40857; RPS6: EOD22278); Bn, *Bigelowiella natans* (S6K: JGI_V11_93000; RPS6: JGI_V11_54140); Gt: *Guillardia theta* (S6K: EKX39801; RPS6: EKX40248); Cp, *Cyanophora paradoxa* (S6K: g24660.t2; RPS6: g3357.t1); Sc, *Saccharomyces cerevisiae* (YPK3: YBR028C; RPS6: YPL090C); and Hs, *Homo sapiens* (S6K: ENST00000225577; RPS6: ENST00000380394). Alignments were performed using the MUSCLE algorithm in MEGA-X.

In the presence of nutrients, mammalian TOR directly phosphorylates the C-terminus of p70 S6 kinase (S6K) at Thr389, which is located at the well-conserved domain FLGFTYVAP. In turn, phosphorylated S6K phosphorylates the C-terminus of the 40S ribosomal protein S6 (RPS6) at conserved Ser residues to activate translation initiation (Fig. 4A). This branch of the TOR pathway is highly conserved from yeast to plants and mammals (Chung *et al.*, 1992; Henriques *et al.*, 2010; Schepetilnikov *et al.*, 2011; Xiong and Sheen, 2012; Dobrenel *et al.*, 2016b), and recent studies support the conservation of this signaling cascade also in microalgae. First, the TOR phosphorylation motif containing the conserved Thr has been identified in the S6K from Chlamydomonas (Fig. 4B), although the size of this protein is controversial, probably due to incorrect annotation of the *S6K* gene (Upadhyaya *et al.*, 2020). Phosphorylation of the conserved Thr at the Chlamydomonas S6K is sensitive to TOR inhibition, indicating that TOR regulates the phosphorylation of this residue (Upadhyaya *et al.*, 2020). Second, phosphorylation of RPS6 at Ser245 strongly decreased upon rapamycin treatment in Chlamydomonas, demonstrating that TOR controls the phosphorylation of this protein (Couso *et al.*, 2020). Third, TOR inhibition results in decreased polysome formation in *C. merolae* (Imamura *et al.*, 2013). Finally, *in vivo* and *in vitro* studies showed that *C. merolae* TOR directly phosphorylates 4E-BP1 (Imamura *et al.*, 2013, 2017), another well-established TOR target in mammals that regulates translation initiation (Gingras *et al.*, 2001). Taken together, these studies demonstrate that TOR regulates protein synthesis in microalgae.

Initial characterization of rapamycin-treated Chlamydomonas cells indicated that TOR negatively regulates autophagy in photosynthetic eukaryotes (Crespo *et al.*, 2005; Perez-Perez and Crespo, 2010; Perez-Perez *et al.*, 2010). Autophagy is a major catabolic pathway that allows eukaryotic cells to degrade and recycle unnecessary or damaged material in order to maintain cellular homeostasis (Michaeli *et al.*, 2016; Mugume *et al.*, 2020; Nakatogawa, 2020). This degradative process is characterized by the formation of double-membrane vesicles known as autophagosomes, which engulf and deliver the cargo to the vacuole. Autophagy is highly conserved in microalgae, with the remarkable exception of red algae, which seem to lack central components of the autophagy machinery (Diaz-Troya *et al.*, 2008b; Shemi *et al.*, 2015). Inhibition of TOR signaling by rapamycin has also been shown to trigger autophagy-like processes in the Haptophyta *Emiliania huxleyi* (Schatz *et al.*, 2014), suggesting that the control of autophagy by TOR is conserved in evolutionarily distant microalgae.

## TOR as a master metabolic regulator in microalgae

Microalgae undergo profound metabolic changes under stress conditions such as nutrient starvation. Stress-induced metabolic reprogramming includes the synthesis and accumulation of carbon storage molecules, mainly triacylglycerols (TAGs) and starch. Remarkably, inhibition of TOR signaling by rapamycin, AZD8055, or Torin1 results in the accumulation of TAGs and starch in divergent groups of microalgae, indicating that algal TOR may play an important regulatory role in the synthesis of these storage molecules. In a rapamycin-sensitive strain of the red alga *C. merolae*, it has been shown that rapamycin treatment promotes the synthesis of TAGs and the formation of lipid droplets to the levels detected under nitrogen starvation (Imamura *et al.*, 2015). Moreover, transcriptomic analyses performed in nitrogen-depleted or rapamycin-treated *C. merolae* cells revealed an up-regulation of genes involved in fatty acid and TAG synthesis, including glycerol-3-phosphate acyltransferase (GPAT) (Imamura *et al.*, 2015), whose activity is required in this microalga for the synthesis of TAGs (Fukuda *et al.*, 2018).

Chemical inhibition of TOR also led to high TAG levels in Chlamydomonas (Imamura *et al.*, 2015, 2016; Juppner *et al.*, 2018) and the diatom *P. tricornutum* (Prioretti *et al.*, 2017), indicating that the control of TAG synthesis is conserved in diverse microalgae. However, the mechanism by which TOR controls lipid synthesis in microalgae is largely unexplored. In Chlamydomonas, TAG synthesis is regulated by inositol polyphosphate (InsP) since the *vip1-1* mutant defective in the InsP kinase VIP1 contains high levels of TAGs (Couso *et al.*, 2016). Moreover, *vip1-1* mutant cells display hypersensitivity to rapamycin, suggesting a link between InsP and TOR signaling in Chlamydomonas (Couso *et al.*, 2016). Whether this connection takes place upstream or downstream of TOR is unknown. Global proteomic and phosphoproteomic approaches revealed that the Chlamydomonas *vip1-1* mutant shows multiple defects in photosynthetic physiology and identified InsPs as key elements in the control of photo-protective mechanisms that may act independently of TOR (Couso *et al.*, 2021).

In addition to lipids, microalgae accumulate starch as storage metabolites under nutrient stress. Starch accumulation following TOR inactivation has been reported in Chlamydomonas (Juppner *et al.*, 2018) and *C. merolae* (Pancha *et al.*, 2019). The mechanism by which TOR regulates starch synthesis in microalgae has been shown in *C. merolae*. A phosphoproteomic study revealed that phosphorylation of GLG1, a glycogenin required for the initiation of starch/glycogen synthesis, is under the control of TOR (Pancha *et al.*, 2019). Specifically, phosphorylation of GLG1 at Ser613 decreased upon TOR inhibition, suggesting that this phosphorylation may control GLG1 activity and thus starch synthesis. This hypothesis is supported by the finding that overexpression of a phosphomimetic GLG1 mutant, in which Ser613 is replaced by aspartic acid, results in a pronounced decrease in the starch content (Pancha *et al.*, 2019). Given the biotechnological potential of microalgae as a source of energy (Merchant *et al.*, 2012; Liu and Benning, 2013), studies connecting TOR with the synthesis and accumulation of TAGs and starch point out this signaling pathway as a good target to enhance biomass and biofuel production in microalgae (reviewed in Prioretti *et al.*, 2020).

Global transcriptomic, metabolomic, and proteomic analyses have investigated the role of TOR in the control of cell growth in microalgae. Despite the different technical approaches, these omic studies established TOR as a master regulator of metabolism in microalgae. Transcriptomic analyses have been reported in *C. merolae* and Chlamydomonas cells treated with rapamycin. These studies revealed an up-regulation of some metabolic processes including amino acid metabolism, TAG synthesis, and autophagy, and the down-regulation of genes involved in cell division and photosynthesis (Imamura *et al.*, 2015; Kleessen *et al.*, 2015). Metabolomic approaches performed in Chlamydomonas cells treated with rapamycin under continuous light (Lee and Fiehn, 2013; Kleessen *et al.*, 2015) or light/dark cycles (Juppner *et al.*, 2018) indicated that TOR inhibition increased the level of primary metabolites and carbon storage molecules such as TAGs and starch. Moreover, the metabolomic analysis performed in synchronized cells also showed redirection of metabolic processes including the synthesis of amino acids, lipids, and starch within minutes of TOR inhibition (Juppner *et al.*, 2018). In this sense, it has been shown that Chlamydomonas cells accumulate large amounts of virtually all amino acids within 5 min of rapamycin treatment (Mubeen *et al.*, 2018). Remarkably, this accumulation of amino acids is triggered by the massive uptake of ammonium and the activation of enzymes involved in nitrogen assimilation such as glutamine synthetase and glutamine oxoglutarate aminotransferase (Mubeen *et al.*, 2018).

Quantitative proteomics and phosphoproteomics have also been used to investigate the effect of inhibiting TOR signaling in microalgae. In Chlamydomonas, a quantitative label-free approach identified potential phosphorylation sites in TOR-related proteins such as Raptor, S6K, and RPS6, and in proteins involved in translation and carotenoid biosynthesis (Werth *et al.*, 2019). Another *in vivo* quantitative phosphoproteomics study performed in Chlamydomonas cells treated with rapamycin identified differentially phosphorylated residues in S6K, RPS6, calcium-regulated kinases, the phosphatase PP2C, and the autophagy protein ATG7 (Roustan and Weckwerth, 2018). Moreover, the same study showed that plastid-localized proteins involved in the Calvin–Benson–Bassham (CBB) cycle, and sulfur-, cysteine-, and methionine-related proteins are down-regulated in rapamycin-treated cells (Roustan and Weckwerth, 2018). A phosphoproteomics analysis carried out in the red microalga *C. merolae* treated with rapamycin also highlighted the relevance of TOR in the control of translation, carbohydrate metabolism, and amino acid synthesis (Pancha *et al.*, 2019). Collectively, these studies uncovered a growing number of potential TOR targets in microalgae connecting TOR signaling with the control of metabolism, although the underlying mechanisms remain largely unknown.

## Regulation of TOR signaling by nutrients in microalgae

Nutrients, particularly amino acids, are confirmed regulators of TORC1 activity in yeast and mammals, but how nutrient availability is transduced to TORC1 in these organisms is not yet fully understood (Gonzalez and Hall, 2017). In plants, significant progress has been made linking nutrients to the regulation of TOR activity (for recent reviews, see Ingargiola *et al.*, 2020; Burkart and Brandizzi, 2021; Li *et al.*, 2021; Liu and Xiong, 2022), which has been largely facilitated by the establishment of TOR kinase assays using S6K and RPS6 as bona fide TOR-regulated targets (Xiong and Sheen, 2012; Dobrenel *et al.*, 2016b). As discussed above, both S6K and RPS6 proteins are conserved in microalgae (Fig. 4), and recent studies in Chlamydomonas have reported reliable TOR kinase assays by monitoring the phosphorylation state of these proteins. Phosphorylation of S6K at the conserved Thr residue of the TOR phosphorylation motif decreased in Chlamydomonas cells treated with rapamycin, indicating that TOR regulates S6K phosphorylation at this position (Upadhyaya *et al.*, 2020). This TOR kinase assay has been useful to demonstrate that TOR activity is modulated by nitrogen and acetate availability in Chlamydomonas (Upadhyaya *et al.*, 2020).

The high conservation of the TOR phosphorylation motif in S6K proteins from divergent microalgae suggests that phosphorylation at this position might be used to monitor TOR activity in other microalgae (Fig. 4B). RPS6 phosphorylation has also been used to analyze TOR activity in different organisms including yeasts, mammals, and plants. Once activated by TOR, S6K phosphorylates Ser residues in the C-terminus of RPS6. Although RPS6 is well conserved among microalgae, the C-terminus of this protein contains a variable number of Ser residues that might be potentially phosphorylated by TOR (Fig. 4C). In Chlamydomonas, the C-terminus of the RPS6 protein is phosphorylated at Ser245 in a TOR-controlled manner (Couso *et al.*, 2020). Indeed, phosphorylation of RPS6 at Ser245 has been used to investigate the regulation of TOR by nutrients in this model microalga.

**Fig. 5.**
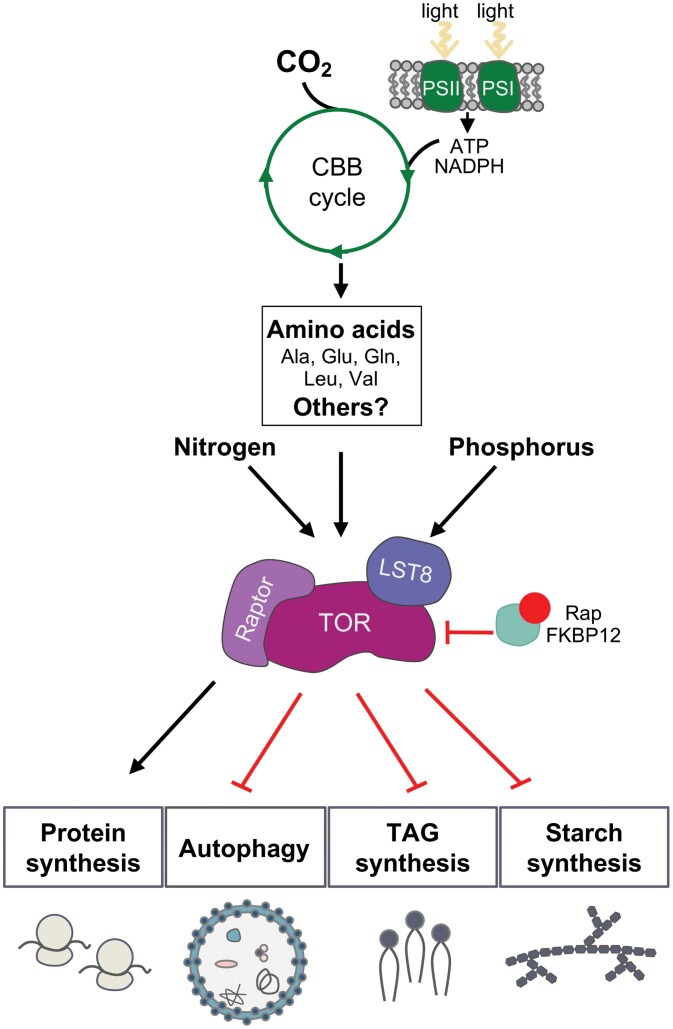
Proposed model for the TOR signaling pathway in the green model microalga *Chlamydomonas reinhardtii*. The TOR kinase forms a high molecular mass complex with LST8 and Raptor similar to TORC1 that can be targeted by rapamycin–FKBP12. TOR activity is controlled by nutrients including phosphorus via LST8 protein abundance (Couso *et al.*, 2020) and nitrogen (Upadhyaya *et al.*, 2020). The photosynthetic assimilation of CO_2_ also activates TOR through the CBB cycle and the synthesis of the central amino acids Ala, Leu, Val, Glu, and Gln (Mallen-Ponce *et al.*, 2022). It is currently unknown whether other metabolites may regulate TOR activity in Chlamydomonas. In the presence of nutrients, TOR is active and promotes cell growth by activating translation and repressing other processes that are usually induced upon nutrient stress such as autophagy and the synthesis of energy storage molecules (TAG and starch).

Phosphorus is an essential nutrient for anabolic processes such as DNA replication, ribosome biogenesis, and translation (Raghothama, 1999), and sensing of phosphorus availability has been recently linked to TOR in Chlamydomonas. Phosphorus starvation inhibits TOR activity by a mechanism that involves the down-regulation of LST8 protein abundance under this nutrient stress (Couso *et al.*, 2020) (Fig. 5). Genetic evidence also supports a link between phosphorus and TOR signaling via LST8. The *lst8-1* knockdown mutant displays a decreased level of LST8 protein and thus TOR activity (Couso *et al.*, 2020). Moreover, TOR activity and LST8 protein abundance are misregulated in a mutant lacking PSR1 (Couso *et al.*, 2020), the main regulator of phosphorus starvation response in both Chlamydomonas and plants (Wykoff *et al.*, 1999; Rubio *et al.*, 2001). Specifically, the *psr1* mutant displayed a lower amount of LST8 protein than wild-type cells under phosphorus sufficiency and failed to down-regulate LST8 abundance and TOR activity under phosphorus limitation (Couso *et al.*, 2020).

TOR has also been linked to the sensing of carbon availability in microalgae. CO_2_ is the primary carbon source for all photosynthetic organisms, and the photosynthetic assimilation of CO_2_ by the CBB cycle strongly regulates TOR activity in Chlamydomonas (Mallen-Ponce *et al.*, 2022). Stimulation of CO_2_ fixation boosts TOR activity, whereas inhibition of the CBB cycle and photosynthesis down-regulates TOR (Mallen-Ponce *et al.*, 2022). Interestingly, the Chlamydomonas starch-deficient mutant *sta6* exhibits extremely high TOR activity, further connecting carbon metabolism with TOR signaling (Mallen-Ponce *et al.*, 2022). In addition to inorganic carbon, reduced carbon in the form of assimilable acetate also activates TOR in Chlamydomonas (Upadhyaya *et al.*, 2020; Mallen-Ponce *et al.*, 2022). The molecular mechanism by which TOR perceives carbon sufficiency in microalgae is unknown. However, it has been shown that the intracellular abundance of the central amino acids in carbon metabolism, Ala, Leu, Val, Gln, and Glu, directly influences TOR activity in Chlamydomonas (Mallen-Ponce *et al.*, 2022) (Fig. 5). In close agreement, mounting evidence indicates that amino acid availability modulates TOR activity in plants (Cao *et al*., 2019; Schaufelberger *et al.*, 2019; O’Leary *et al.*, 2020; Liu *et al.*, 2021).

Photosynthesis provides ATP and NADPH required for CO_2_ fixation, which in turn promotes TOR activity. Therefore, it is plausible that photosynthesis regulates TOR. Supporting this hypothesis, it has been reported that inhibition of photosynthesis down-regulates TOR activity in both algae (Mallen-Ponce *et al.*, 2022) and plants (Riegler *et al.*, 2021). Although these studies clearly position TOR downstream of photosynthesis, inhibition of TOR signaling in microalgae has been shown to regulate photosynthesis and other processes in the chloroplast. Chemical inhibition of TOR by AZD8055 impairs the maintenance of PSI, PSII efficiency, and inhibits state transitions between PSII and PSI in Chlamydomonas (Ford *et al.*, 2019; Upadhyaya and Rao, 2019). Proteomic and phosphoproteomic analyses performed in rapamycin-treated Chlamydomonas cells showed a decrease in most of the proteins involved in the CBB cycle (Roustan and Weckwerth, 2018). Quantitative proteomics of TOR inhibition via enrichment of reversibly oxidized Cys residues also revealed a link between photosynthesis and TOR (Ford *et al.*, 2019). Moreover, a recent phosphoproteomics study showed a particular enrichment for PSII proteins in the Chlamydomonas *vip1-1* mutant following rapamycin treatment, suggesting a role for InsP in governing PSII and photo-protection (Couso *et al.*, 2021). TOR has also been connected to chloroplast function in the red alga *C. merolae*. TOR inhibition results in increased expression of the chloroplastic protein RSH4b, which synthesizes guanosine 3ʹ-diphosphate 5ʹ-diphosphate (ppGpp). The up-regulation of the ppGpp level inhibits rRNA synthesis, leading to decreased protein synthesis in the chloroplast (Imamura *et al.*, 2018). Furthermore, transcription of nuclear and mitochondrial rRNA was also inhibited in *C. merolae* following TOR inactivation, suggesting a possible regulation of ribosome biogenesis in different cellular compartments by TOR (Imamura *et al.*, 2018).

## Perspectives and open questions

The biotechnological use of microalgae as a source of biofuels and value-added products demands a better knowledge in these organisms about the signaling pathways and proteins that regulate cell growth and metabolism such as TOR. However, we are just starting to elucidate the function of TOR in microalgae, and most of our knowledge in this field comes from a few model species, which certainly narrows down the high potential associated with the evolutionary divergence of these organisms. The main regulators of TORC1 signaling in yeasts and mammals such as Rheb, RAGs, or Ragulator are not conserved in microalgae, raising the question of whether specific regulatory mechanisms and proteins evolved in these organisms. Building on this hypothesis, TORC2 seems to be absent in microalgae, although the presence of a TOR complex functionally similar to TORC2 constituted by highly divergent proteins compared with their yeast and mammalian counterparts cannot be ruled out. The answer to this question will probably come with the identification of TOR-interacting proteins in microalgae.

TOR governs important cellular processes including translation, autophagy, and the synthesis of energy storage molecules. How TOR coordinately controls anabolic and catabolic processes and the specific underlying mechanisms are largely unknown in microalgae. Significant progress has been made in connecting TOR with starch and lipid synthesis in *C. merolae* (Imamura *et al.*, 2015; Pancha *et al.*, 2019), but future research should focus on the identification of downstream TOR targets in microalgae. The regulation of TOR signaling by nutrients is also an emerging field in microalgae. The development of TOR kinase assays in Chlamydomonas (Couso *et al.*, 2020; Upadhyaya *et al.*, 2020) has significantly contributed to show that essential nutrients such as CO_2_, nitrogen, and phosphorus regulate TOR activity in microalgae (Couso *et al.*, 2020; Upadhyaya *et al.*, 2020; Mallen-Ponce *et al.*, 2022). Despite the fact that some mechanisms connecting nutrient sufficiency have been partially identified (Couso *et al.*, 2020; Mallen-Ponce *et al.*, 2022), the precise processes by which TOR perceives the availability of different nutrients in microalgae are still largely unknown. Finally, whether TOR coordinates with other nutrient signaling pathways such as the highly conserved AMPK/Snf1/SnRK1 to regulate cell growth has not been explored yet in microalgae although the well-defined crosstalk of these pathways in mammals (Gonzalez *et al.*, 2020) and plants (Belda-Palazon *et al.*, 2020) points to a similar connection in microalgae.

## Abbreviations

CBB: Calvin–Benson–Bassham cycle
LECA: last eukaryotic common ancestor
TAG: triacylglycerol
TORC1: TOR complex 1.
